# Effects of intravenous propofol combined with opioids on euphoria in patients undergoing painless gastrointestinal endoscopy: protocol for a randomized double-blind placebo-controlled trial

**DOI:** 10.3389/fphar.2025.1599684

**Published:** 2025-05-30

**Authors:** Youjia Yu, Qingfang Ma, Fengxia Du, Xiang Zhang, Chunyan Shao, Yan Li, Chuntao Ma

**Affiliations:** ^1^ Department of Pain and Anesthesiology, Suzhou Xiangcheng People’s Hospital, Suzhou, China; ^2^ Department of Gastroenterology and Endoscopy Center, Suzhou Xiangcheng People’s Hospital, Suzhou, China; ^3^ Department of Nursing, Suzhou Xiangcheng People’s Hospital, Suzhou, China

**Keywords:** propofol, opioids, fentanyl, nalbuphine, gastrointestinal endoscopy, euphoria

## Abstract

**Background:**

In recent years, the rapid increase in gastrointestinal endoscopic procedures has posed new demands and challenges for painless and comfortable medical care. Intravenous anesthetics may increase postoperative euphoria in patients. This study aims to evaluate the effects of intravenous propofol combined with fentanyl, nalbuphine, or saline on euphoria in patients undergoing painless gastrointestinal endoscopy.

**Methods:**

This is a single-center, randomized, double-blind, placebo-controlled trial protocol involving 285 adult patients scheduled for bidirectional endoscopy. Participants will be randomly assigned to either the fentanyl group, the nalbuphine group, or the placebo group (n = 95 per group). The fentanyl group will receive propofol + fentanyl; the nalbuphine group will receive propofol + nalbuphine; and the placebo group will receive propofol + saline. The primary outcome is the Addiction Research Center Inventory (ARCI) - Morphine–Benzedrine Group (MBG) scores at 30 min post-awakening. Secondary outcomes will include ARCI-MBG scores at 1 week and 1 month postoperatively; ARCI total scores at 30 min post-awakening, 1 week, and 1 month postoperatively; ARCI-Pentobarbital, Chlorpromazine, Alcohol Group (PCAG) scores at 30 min post-awakening, 1 week, and 1 month postoperatively; ARCI-Lysergic Acid Diethylamide (LSD) scores at 30 min post-awakening, 1 week, and 1 month postoperatively; Pittsburgh Sleep Quality Index scores at 1 week and 1 month postoperatively; and dream descriptions (none, pleasant, or nightmare) at 30 min post-awakening; The Surgical Pleth Index assessed at the time of endoscope insertion. Safety outcomes will include desaturation, hypotension, nausea or vomiting, dizziness, headache, choking cough, involuntary movement, bradycardia, and airway intervention. Data will be analyzed following a modified intention-to-treat approach.

**Discussion:**

This study aims to provide high-quality evidence for the potential addictive properties and safety of propofol combined with opioids in gastrointestinal endoscopy procedures.

**Ethics and Dissemination:**

This study was approved by the Ethics Committee of Xiangcheng People’s Hospital of Suzhou (2024-KY-05) on 19 August 2024, and is registered with the Chinese Clinical Trial Registry (ChiCTR2500096595). All participants will provide written informed consent, and the study will adhere to the principles of the Declaration of Helsinki. The findings will be published in a peer-reviewed journal. URL: https://www.chictr.org.cn/showproj.html?proj=252367.

## 1 Introduction

Gastrointestinal endoscopy is an essential method for diagnosing and treating gastrointestinal diseases, with a large number of endoscopic examinations performed globally each year. In China, a national survey projects that, driven by an aging population, the number of painless gastrointestinal endoscopies will reach approximately 51 million by 2030 (Zhou et al., 2021). Two-way endoscopy, where esophagogastroduodenoscopy and colonoscopy are conducted simultaneously in a single visit, is gaining increasing acceptance among patients (Song et al., 2023; Choi et al., 2020). To enhance patient comfort and reduce the influence of patient movement on the quality and safety of endoscopic procedures, the use of sedatives is becoming increasingly common (Dossa et al., 2021). Among the available sedatives, propofol, recognized for its rapid onset and recovery, has become one of the most widely used agents for painless gastrointestinal endoscopy worldwide (Lucendo et al., 2015; Goudra, 2019). Although severe respiratory and cardiovascular complications with propofol are relatively infrequent, both animal models and healthy volunteer studies have demonstrated its capacity to induce euphoria and suggested its possible involvement in dopaminergic signaling within the ventral tegmental area (VTA) and nucleus accumbens (NAc) (Nishizawa and Suzuki, 2018; Brechmann et al., 2018). Propofol has been shown to produce rewarding and reinforcing effects in animal studies, with evidence of self-administration and conditioned place preference, indicating its potential for abuse (Roussin et al., 2007). In humans, clinical trials reveal that propofol can induce euphoria and subjective feelings of pleasure, further supporting its potential for recreational use and addiction (Roussin et al., 2007). Consequently, these findings have raised concerns about propofol’s potential for drug dependence and addiction risk. In recent years, the increase in reported cases of propofol abuse has become a concerning trend, prompting some researchers to advocate for classifying propofol as a controlled substance (Uzbay and Shahzadi, 2024).

Propofol is widely used for sedation in various surgical patients, gastrointestinal endoscopic procedures, outpatient minor surgeries, and for sedation in ICU patients (McKeage and Perry, 2003; Hewson et al., 2021). Propofol, whether administered alone or in combination with opioid analgesics such as fentanyl or nalbuphine, is a common sedation strategy in digestive endoscopy (Sahinovic et al., 2018; Dossa et al., 2020). Fentanyl, a potent synthetic opioid, exerts its analgesic effects by activating μ-opioid receptors and enhancing dopamine release in the ventral tegmental area and nucleus accumbens—processes that induce both rewarding sensations and euphoria—and it also carries a risk of drug abuse (Kreek et al., 2019). Given fentanyl’s inherent ability to evoke euphoria, we hypothesize that its co-administration may further augment propofol’s euphoric effect. In contrast, nalbuphine, a mixed agonist–antagonist that primarily acts as a κ-opioid receptor agonist and partially antagonizes μ-opioid receptors, rarely causes euphoria and displays minimal drug-seeking behavior or physiological dependence, suggesting a low potential for abuse (Raghav et al., 2018). Accordingly, we propose that nalbuphine’s partial μ-opioid receptor antagonistic properties might help mitigate propofol’s excitatory or euphoric effects. Gastrointestinal endoscopy is widely performed and frequently repeated (Akarsu Ayazoglu and Uzman, 2021), yet there remains a lack of comprehensive clinical research examining whether co-administration of propofol with different opioids results in distinct euphoria-related responses. This study aims to investigate whether propofol co-administered with fentanyl or nalbuphine differentially influences euphoria in patients undergoing gastrointestinal endoscopy. By thoroughly evaluating the impact of these sedation strategies, we hope to provide valuable insights and potentially set a new standard for painless endoscopic procedures, thereby improving patient safety and overall outcomes.

## 2 Methods

This protocol adheres to the Standard Protocol Items: Recommendations for Interventional Trials (SPIRIT) guidelines.

### 2.1 Study design and patients

**FIGURE 1:**
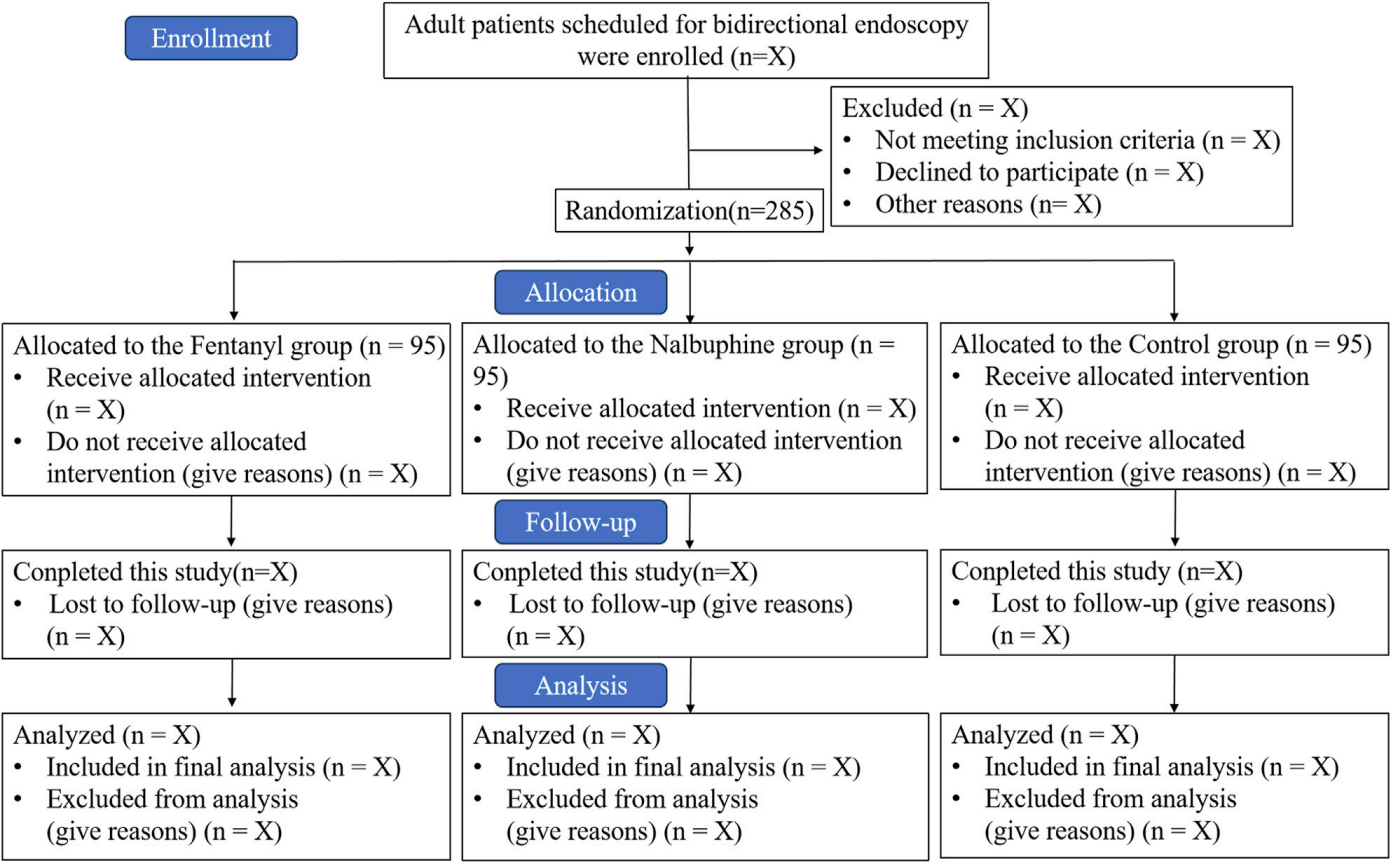
Study flow diagram. Intervention: Three groups were administered 0.5 μg/kg of intravenous propofol along with either 0.03 mg of fentanyl, 3 mg of nalbuphine, or an equivalent volume of saline to induce sedation. Throughout the procedures, sedation was adjusted by titrating propofol doses, typically ranging from 0.2 to 0.3 mg/kg, to maintain the predetermined sedation level.

This study is a single-center, prospective, randomized, double-blind, placebo-controlled, parallel-group clinical trial. The trial will be conducted at the Suzhou Xiangcheng People’s Hospital, enrolling a total of 285 patients. Recruitment is scheduled to take place from 15 February 2025 to 31 August 2025. The study flow diagram is shown in Figure 1.

### 2.2 Inclusion criteria

Patients who meet the following criteria will be included:

• Aged from 18 to 75 years old;
• Body mass index (BMI) 18–30 kg/m^2^;
• ASA grade I-III;
• Mallampati grade I-III;
• Patients undergoing bidirectional endoscopy
• Patients can understand the whole process of this clinical study and sign the informed consent.

### 2.3 Exclusion criteria

The exclusion criteria include:

• Complicated with serious heart, lung or brain diseases;
• Patients with severe sleep apnea syndrome;
• Patients with severe esophageal and gastric motility diseases;
• Long-term use of opioids or hypnotics and significant dependence on sedative or analgesic drugs. Long-term opioid use;
• Patients allergic to propofol, fat emulsion, soy, or egg;
• Pregnant or lactating women;
• Language communication disorder, lack of cooperation, inability to communicate, or mental disease;
• Mallampati grade IV, mouth opening < 2.5 cm; Patients who might have or had a history of difficult airway or had a history of abnormal anesthesia recovery were predicted;
• Chronic alcoholics

### 2.4 Primary outcome

The primary outcome will be the Addiction Research Center Inventory (ARCI) - Morphine–Benzedrine Group (MBG) scores, assessed via in-person interviews at 30 min post-awakening. The ARCI scale was developed by the National Institute of Mental Health Addiction Research Center (United States). The MBG, a subscale of the ARCI, measures drug-induced euphoria and helps assess whether patients experience euphoria. The MBG consists of items 1 to 16, and the sum of these items represents the euphoria score. The Chinese version used in this study was translated by experts, with its reliability and validity confirmed.

### 2.5 Secondary outcomes

Secondary outcomes will include ARCI-MBG scores at 1 week and 1 month postoperatively; ARCI total scores at 30 min post-awakening, 1 week, and 1 month postoperatively; ARCI-Pentobarbital, Chlorpromazine, Alcohol Group (PCAG) scores at 30 min post-awakening, 1 week, and 1 month postoperatively; ARCI-Lysergic Acid Diethylamide (LSD) scores at 30 min post-awakening, 1 week, and 1 month postoperatively; Pittsburgh Sleep Quality Index scores at 1 week and 1 month postoperatively; and dream descriptions (none, pleasant, or nightmare) at 30 min post-awakening; The Surgical Pleth Index assessed at the time of endoscope insertion.

### 2.6 Safety outcomes

Safety outcomes will desaturation, hypotension, nausea or vomiting, dizziness, headache, choking cough, involuntary movement, bradycardia, and airway intervention.

### 2.7 Randomization and blinding

An independent researcher will generate random numbers using an online tool (https://www.sealedenvelope.com/simple-randomiser/v1/lists) with a 1:1:1 allocation ratio. The randomization results will be stored in sealed, opaque envelopes. Patients will be assigned to the fentanyl, nalbuphine, or placebo group. A nurse anesthesiologist not involved in other study procedures will prepare fentanyl, nalbuphine, and saline in syringes labeled only with patient numbers, which will also be marked on the outer packaging. Patients, surgeons, anesthesia providers, outcome assessors, and statisticians will all be blinded to group assignments.

### 2.8 Study interventions

In this study, patients will receive intravenous propofol combined with either fentanyl, nalbuphine, or saline during painless gastrointestinal endoscopy. Patients in the fentanyl group will receive a combination of propofol and fentanyl, those in the nalbuphine group will receive propofol and nalbuphine, and the placebo group will receive propofol with saline. All patient groups were administered 0.5 μg/kg of intravenous propofol along with either 0.03 mg of fentanyl, 3 mg of nalbuphine, or an equivalent volume of saline to induce sedation. Throughout the procedures, sedation was adjusted by titrating propofol doses, typically ranging from 0.2 to 0.3 mg/kg, to maintain the predetermined sedation level. At the start of esophagogastroduodenoscopy, the target sedation level was set at a Modified Observer’s Assessment of Alertness/Sedation (MOAA/S) scale score of 1 (responding only to trapezius squeeze stimulus). During the subsequent colonoscopy, the target sedation level was set at a score of 2 (responding only to prodding or shaking stimuli). All medications will be administered by medical professionals who are not involved in patient recruitment or data collection to maintain blinding. Randomization will be performed prior to the start of the study by an independent researcher using a computer-generated randomization table, and group assignments will be stored in sealed opaque envelopes to ensure blinding of patients, investigators, and data analysts. The study schedule for patient enrollment, interventions, and outcome assessments will follow the SPIRIT statement (Table 1). All endoscopy procedures will be performed by experienced endoscopists who have conducted at least 1,000 gastrointestinal endoscopies procedures to the study.

**TABLE 1:** Schedule of patient enrolment, study interventions and outcome assessment.

Time point	Study period						
	Enrolment	Allocation	Post-allocation			Follow-up	Follow-up
	Pre-op visit	Pre-op	Intra-op	Post-op	30 min post-awake	1 week	1 month
Patient enrolment							
Eligibility criteria	×						
Written informed consent	×						
Demographic data	×						
Baseline characteristics	×						
HADS	×						
Randomization/allocation		×					
Study interventions							
Propofol + fentanyl		×					
Propofol + nalbuphine		×					
Propofol + saline		×					
Outcome assessment							
ARCI-MBG					×	×	×
ARCI-PCAG					×	×	×
ARCI-LSD					×	×	×
ARCI-Total					×	×	×
PSQI						×	×
Dream descriptions					×		
SPI			×		×	×	×
Time of induction		×			×	×	×
Time to recovery				×			×
Time awake				×			×
Endoscopists highly satisfied				×			
Patients highly satisfied				×			
Desaturation			×	×			
Hypotension			×	×			
Nausea or vomiting			×	×			
Dizziness			×	×			
Headache			×	×			
Choking cough			×				
Involuntary movement			×				
Bradycardia			×	×			
Airway intervention			×	×			
Spasm of the airway			×	×			

According to SPIRIT, statement of defining standard protocol items for clinical trials.

Pre-op, Preoperative; Intra-op, Intraoperative; Post-op, Postoperative; HADS, hospital anxiety and depression scale; ARCI, addiction research center inventory; MBG, Morphine–Benzedrine Group; PCAG, pentobarbital, Chlorpromazine, Alcohol Group; LSD, lysergic acid diethylamide; PSQI, pittsburgh sleep quality index; SPI, surgical pleth index; SPIRIT, Standard Protocol Items: Recommendations for Interventional Trials.

### 2.9 Anaesthetic care

Venous access was established immediately after the patient entered the examination room, and vital signs were monitored. Intraoperative monitoring included non-invasive blood pressure (NIBP), electrocardiography (ECG), and pulse oximetry (SpO_2_). Oxygen was administered via a nasal cannula at a flow rate of 3 L/min until patients completed the gastroscopy and were fully awake. Anesthesia for the three groups of patients was induced with an intravenous injection of propofol at 1.5–2.5 mg/kg, combined with either fentanyl (0.05 mg), nalbuphine (5 mg), or an equivalent volume of saline. Sedation was adjusted throughout the procedures by titrating propofol doses, typically 0.2–0.3 mg/kg, to achieve the desired sedation level. At the start of esophagogastroduodenoscopy, the target sedation level was set at a Modified Observer’s Assessment of Alertness/Sedation (MOAA/S) scale score of 1 (responsive only to a trapezius squeeze stimulus). During the subsequent colonoscopy, the target sedation level was adjusted to a score of 2 (responsive only to prodding or shaking). Hypotension, defined as a systolic blood pressure of <80 mm Hg or a 30% decrease from baseline, and bradycardia, defined as a heart rate <45 beats/min, were treated as necessary. If SpO_2_ fell below 90% for 10 s or more, supplemental oxygen at 5–10 L/min was administered, and airway interventions, such as jaw thrust, oral or nasal airway placement, or endotracheal intubation, were performed as needed.

### 2.10 Data collection and monitoring

Data collection will include baseline characteristics such as age, sex, BMI, comorbidities, smoking status, alcohol consumption, education level, and scores from the Hospital Anxiety and Depression Scale (HADS), as well as the number of painless examinations. All data will be documented in case report forms (CRFs) and subsequently entered into the electronic database under the supervision of the principal investigator. An independent Data Monitoring Committee (DMC) will continuously monitor the data collection process. Once data registration is complete, the electronic database will be secured. De-identified datasets will be provided to an independent statistician for analysis based on a predefined plan. Serious adverse events (SAEs), related or unrelated to the study medication (e.g., persistent severe psychiatric symptoms), must be promptly reported to the principal investigator. The perioperative care team will take necessary actions to ensure participant safety, and these events must be reported to the DMC within 24 h for further evaluation and possible adjustments or study termination. To ensure the integrity and accuracy of the collected data, we implemented the following measures: A double-check mechanism for data entry, where all data entries are verified by a second independent researcher; Regular audits of the collected data to identify and correct any inconsistencies or errors; Standardized training for all researchers involved in data collection to ensure consistent protocols and reduce the risk of errors.

### 2.11 Sample size calculation

Based on the previous study (Li et al., 2024), the mean ARCI-MBG score 30 min after patients wake from gastrointestinal endoscopy is 8.84 with a standard deviation of 2.22. To detect a difference of 0.5 standard deviations between groups, and using the Bonferroni correction with a significance level of 0.017% and 80% power, we calculated a sample size of 95 patients per group. Considering a 10% dropout rate, the total required sample size is 285 patients, assuming a two-sided α of 0.05% and 80% power.

To calculate the sample size, we used the following formula for two independent groups with a normal distribution: n=(Z_1−α′/2_+Z_1−β_)^2^ × 2/d^2^.

Where: Z_1−α′/2_ is the critical value for a two-sided α′ of 0.017 (2.396), Z_1−β_ is the critical value for a power of 80% (0.842), d is 0.5. Using these values and applying a Bonferroni correction for multiple comparisons (α′ = 0.017).

### 2.12 Statistical analysis

The normality of continuous variables will be evaluated using the Shapiro-Wilk test. Variables following a normal distribution will be reported as mean (standard deviation), while non-normally distributed variables will be expressed as median (interquartile range). The continuous data of normal distribution were analyzed by 1-way analysis of variance, and the continuous data of nonnormal distribution among the three groups were analyzed by the Kruskal-Wallis rank-sum test. Categorical data were analyzed using the χ^2^ test or Fisher’s exact test, and the P value was adjusted according to Bonferroni method and fixed at 0.017 for pairwise comparison. P < 0.05 was considered to indicate significance.

Outcome analyses for the primary endpoint will primarily follow a modified intention-to-treat (mITT) approach, including all participants for whom relevant data are available, regardless of protocol adherence. Additionally, a per-protocol (PP) analysis will be conducted, excluding participants with protocol deviations or those who withdrew consent, to evaluate the treatment effect among those who strictly followed the study protocol. The primary outcome is the ARCI-MBG score measured 30 min post-awakening. To account for potential confounders, a covariate-adjusted linear mixed model (LMM) will be utilized. The model will incorporate fixed effects for group (treatment group), time points (30 min post-awakening, 1 week, and 1 month), and the interaction between group and time to evaluate differences across groups over time. Potential confounders, including HADS scores, sex, age, BMI, comorbidities, smoking status, alcohol consumption, education level, and the number of painless examinations, will be included as covariates in the model. Sensitivity analyses will be conducted using both mITT and PP approaches to evaluate the robustness of the findings. Additionally, these analyses will adjust for potential confounders such as sex, age, BMI, comorbidities, smoking status, and education level, to further assess the consistency of the results across different analytical methods. No interim analysis will be planned. Missing data will not be imputed. Statistical analyses will be conducted with the use of SPSS software (V.25.0; IBM SPSS).

### 2.13 Patient and public involvement

Patients and the public will not be involved in the design, recruitment, conduct, or reporting of this study. The study results will be communicated to participants through email.

### 2.14 Principles and methods of unblinding or breaking the blind

#### 2.14.1 Unblinding timeline

Participants will be unblinded after the study concludes, once all subjects have completed the 1-month follow-up period. Unblinding Method: An independent DMC will manage the unblinding process, overseeing and securely retaining all randomization data until the scheduled unblinding.

#### 2.14.2 Emergency unblinding

In the event of a serious adverse event (SAE) or other emergency during the trial, unblinding will be conducted immediately to ensure appropriate medical intervention. The principal investigator will request emergency unblinding from the DMC, with the reasons and process for unblinding fully documented.

## 3 Discussion

This study is a single-center, randomized, double-blind, placebo-controlled trial involving 285 adult patients scheduled to undergo bidirectional gastrointestinal endoscopy. The primary objective is to evaluate the differential effects of intravenous propofol administration combined with fentanyl, nalbuphine, or normal saline on patient-reported euphoria during painless endoscopy. The primary outcome measure is the Morphine-Benzedrine Group (MBG) subscale score of the Addiction Research Center Inventory (ARCI), which assesses drug-induced euphoria. ARCI is a validated psychometric instrument developed by the National Institute of Mental Health Addiction Research Center, comprising MBG, PCAG, and LSD subscales that assess drug-induced euphoria, sedation, and anxiety, respectively. Specifically, the MBG subscale quantifies euphoric effects and helps determine whether patients experience significant euphoria. Secondary outcomes, including sedation and anxiety, will also be evaluated systematically at 30 min, 1 week, and 1 month after the procedure through questionnaires and ARCI scores, and related influencing factors will be analyzed statistically. This study will be conducted in accordance with the CONSORT guidelines. The trial will be conducted and reported in accordance with the Consolidated Standards of Reporting Trials (CONSORT) guidelines. To control for potential confounding effects and ensure the validity of the primary results, baseline covariates will be adjusted using appropriate statistical methods. Additionally, sensitivity analyses will be performed to assess the robustness and consistency of the findings.

Propofol is a γ-aminobutyric acid (GABA) receptor agonist that enhances the function of GABA type-A receptors (GABA_AR) on the postsynaptic membrane, thereby exerting sedative, anxiolytic, and antiepileptic effects (Yang et al., 2011). It is currently among the most commonly administered intravenous anesthetics in clinical practice worldwide (Baker and Naguib, 2005; Trapani et al., 2000; Solomon et al., 2019). Due to its rapid onset and short half-life, propofol has become particularly favored for sedation during gastrointestinal endoscopy (Walsh, 2022). However, recent increases in reported cases of propofol addiction have raised significant concern among both healthcare providers and the broader community. Clinical studies have indicated that more than half of patients receiving propofol report subjective sensations of relaxation, pleasure, or euphoria (Kim et al., 2013; Brechmann et al., 2018). Animal studies further support these findings, demonstrating that rats can reliably distinguish propofol from control solvents and exhibit reward-associated behaviors, suggesting propofol’s potential for addiction (Zacny et al., 1993; Shahzadi et al., 2018). Emerging evidence suggests that the mesolimbic-cortical pathway—particularly the neuronal circuitry linking the ventral tegmental area (VTA) to the nucleus accumbens (NAc) and prefrontal cortex—as well as various neurotransmitters and their corresponding receptors, plays a critical role in mediating the euphoric and rewarding effects induced by propofol (Dong et al., 2021; Chen et al., 2020; Yang et al., 2011).

During gastrointestinal endoscopy, sedation with propofol combined with fentanyl is a commonly used clinical regimen (Luginbuhl et al., 2009). Fentanyl, a potent synthetic opioid analgesic approximately 50–100 times more potent than morphine, has recognized addictive potential and induces pronounced euphoria through activation of the μ-opioid receptor (MOR) and associated reward pathways (Vardanyan and Hruby, 2014; Volkow et al., 2019). Given these pharmacological characteristics, fentanyl may modulate patients’ subjective responses to propofol, potentially intensifying its euphoric effects.

Nalbuphine is a mixed opioid agonist-antagonist characterized by its dual pharmacological effects: κ-opioid receptor agonism and μ-opioid receptor antagonism (Errick and Heel, 1983). Compared with traditional opioids such as morphine and fentanyl, nalbuphine primarily achieves analgesia through κ-receptor activation, while its antagonistic effect on μ-receptors significantly reduces associated euphoria, respiratory depression, and risk of dependence (Ibrahim et al., 2018; Hammond, 1984; Jannuzzi, 2016). Due to these pharmacological characteristics, nalbuphine has a notably lower abuse potential and has been increasingly used in endoscopic sedation in recent years (Li S et al., 2021). Considering nalbuphine’s antagonistic action on μ-receptors—key mediators of opioid-induced euphoria—it is plausible to hypothesize that when combined with propofol, nalbuphine might attenuate or counteract propofol-induced euphoric effects, potentially offering clinical benefits in sedation strategies aimed at reducing drug dependence risk.

We note that missing data will not be imputed. The rationale behind this approach is to avoid introducing potential bias by making assumptions about missing values, particularly in the absence of a clear pattern of missingness. Furthermore, since most of our data were collected in a hospital setting, the missing data represent only a very small fraction of the overall dataset. Given that the missing data are unlikely to be systematically related to key variables, we believe that imputation could introduce unnecessary assumptions that may not accurately reflect the true data distribution.

This study has several limitations. First, as a single-center trial, the generalizability of the results to broader clinical settings may be limited; multi-center studies involving diverse patient populations would be necessary to validate these findings. Second, the relatively short follow-up duration (1 month) restricts the evaluation of potential long-term euphoric effects and addiction risks associated with repeated exposure to propofol and opioid combinations. Extended follow-up periods would provide deeper insights into the chronic implications of these sedation regimens. Third, the exclusion criteria—especially excluding patients with long-term opioid or sedative usage—may introduce selection bias, limiting the external validity of the results. Including such patient groups in future research could yield a more comprehensive understanding of drug-related addictive risks. Fourth, the primary outcomes of this study rely on subjective self-reported measures of euphoria, which might be susceptible to response bias; incorporating objective biomarkers or neuroimaging techniques in future studies could enhance the objectivity of the findings. Lastly, the current study design does not include objective behavioral assessments of addiction-related behaviors, such as drug-seeking actions, thereby limiting the evaluation of true addiction potential. Future investigations should explore other sedative drug combinations and their potential effects on euphoria, as well as further examine objective behavioral events related to addiction.

The primary aim of this randomized, double-blind, placebo-controlled trial is to evaluate the differential effects of intravenous propofol combined with fentanyl, nalbuphine, or placebo on patient-reported euphoria following painless gastrointestinal endoscopy. By systematically examining the impact of these sedation regimens, the study seeks to determine whether fentanyl amplifies, or nalbuphine mitigates, propofol-induced euphoric sensations. The findings are anticipated to provide robust clinical evidence to guide sedation strategies, potentially optimizing postoperative sedation protocols and enhancing patient safety and outcomes in gastrointestinal endoscopic practice. Additionally, if the results confirm that nalbuphine reduces euphoria compared to fentanyl, this could lead to safer sedation protocols by minimizing the risk of addiction-related side effects.
